# Immune and Vascular Function in Cardiometabolic Disorders: Interplay With Sex Differences and Impact on Incretin Therapy

**DOI:** 10.1111/apha.70091

**Published:** 2025-08-21

**Authors:** Anirudh Subramanian Muralikrishnan, Valentina Biasin, Diana Zabini, Elena Osto

**Affiliations:** ^1^ Division of Physiology and Pathophysiology, Otto Loewi Research Center for Vascular Biology, Immunology and Inflammation Medical University of Graz Graz Austria; ^2^ Lung Research Cluster, Otto Loewi Research Center for Vascular Biology, Immunology and Inflammation Medical University of Graz Graz Austria; ^3^ Vetsuisse Faculty University of Zurich Zurich Switzerland

**Keywords:** endothelial cells, GIP, GLP‐1, immune cells, incretins, sex differences

## Abstract

**Background and Aims:**

Vascular dysfunction, driven by endothelial impairment, arterial stiffness, inflammation, and immune activation, contributes to cardiometabolic disorders such as hypertension and atherosclerosis. Sex differences and sex hormones influence the progression of vascular and immune dysfunction. Incretin hormones, including glucagon‐like peptide‐1 (GLP‐1) and glucose‐dependent insulinotropic polypeptide (GIP), regulate glucose homeostasis and also impact vascular and immuno‐metabolic health. This review examines their roles in these processes, with emphasis on sex‐specific effects.

**Methods:**

A narrative review of preclinical and clinical studies assessing GLP‐1 and GIP actions on vascular function, immune regulation, and metabolism, and their modulation by sex and sex hormones.

**Results:**

Incretins improve endothelial function, reduce vascular inflammation, and modulate immune‐metabolic crosstalk, processes often impaired in cardiometabolic disease. Sex differences affect incretin secretion, signalling, and therapeutic responses, though underlying mechanisms remain unclear.

**Conclusions:**

Incretin hormones are promising targets for improving vascular and immune‐metabolic health in cardiometabolic disorders. Understanding sex‐specific mechanisms will be essential for optimizing incretin‐based therapies.

## 1 Introduction

Cardiometabolic disease (CMD) encompasses the interplay between metabolic dysfunction (such as obesity, dyslipidemia and diabetes), atherosclerotic cardiovascular disease, and its complications [1]. The development of CMD is influenced by multifaceted risk factors, including age [2], genetic predisposition [3], environmental factors like poor diet and physical inactivity, and hormonal disruptions such as insulin resistance [4]. CMDs share key pathological molecular mechanisms, including chronic inflammation and endothelial dysfunction [5]. Indeed, endothelial dysfunction driven by metabolic changes is considered one of the first hallmarks of CMD leading to impaired blood flow, hypertension, and atherosclerosis [6]. A systemic chronic low‐grade inflammation, driven by cellular metabolic alterations in multiple immune cell subsets, is observed throughout the different stages of CMD [7, 8]. Incretin hormones, primarily glucagon‐like peptide‐1 (GLP‐1) and gastric inhibitory polypeptide (GIP), are gut‐derived peptides that regulate post‐prandial blood glucose levels by promoting insulin release and by inhibiting glucagon secretion in response to food intake [9, 10]. Incretin‐based therapies using single agonists towards GLP‐1 receptor (GLP‐1R) or GIP receptor (GIPR) and dual agonists towards GLP‐1R/GIPR have proven to be effective therapeutic options in type 2 diabetes (T2D), obesity, and related CVDs [11, 12]. However, incretin and incretin‐based therapies exert pleiotropic effects on the heart [13], brain [14, 15], muscles, and adipose tissue [16]. Strong evidence indicates that these therapies offer cardiovascular benefits by improving endothelial dysfunction and reducing systemic inflammation [17, 18].

This review provides an overview of vascular and immune functions and emerging evidence on their interaction with incretin hormones and analogs. Physiological and CMD conditions will be reviewed, particularly in relation to obesity, T2D, and associated cardiovascular risk factors such as hypertension and atherosclerosis. The discussion of molecular mechanisms will include a focus on sex differences, emphasizing the importance of incorporating sex‐dependent factors in CMD research. Sex will be considered as a biological variable, influenced by endocrine, genetic, and anatomical factors, while gender, shaped by social, economic, and political influences, will not be the primary focus of this review [19, 20].

## 2 Molecular Mechanisms in Physiology and CMD

### 2.1 Endothelial Function

**FIGURE 1:**
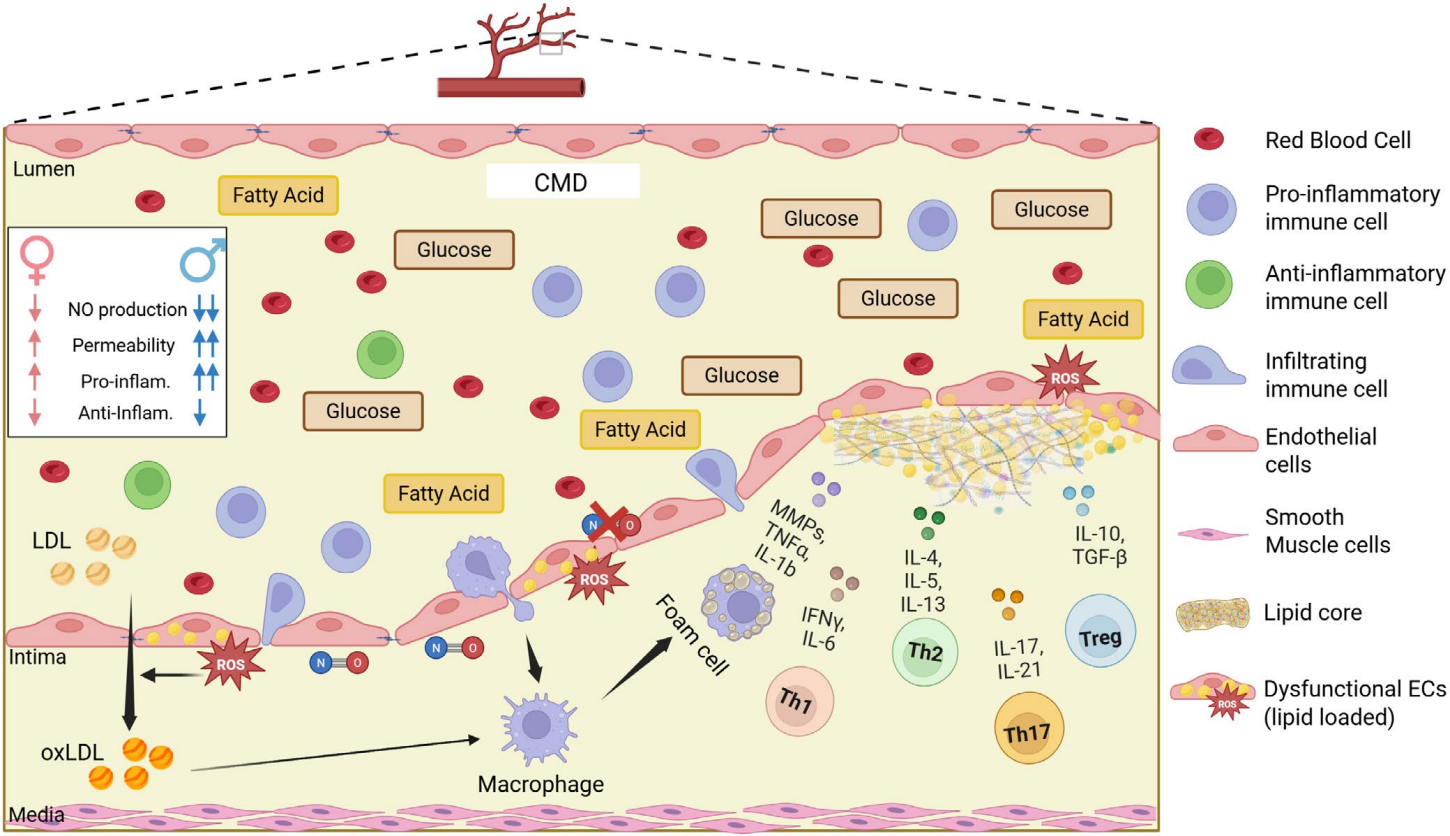
Graphic representation of the endothelial and immune cell dysfunction in atherosclerotic CMD. Increased circulating glucose and fatty acids might affect both immune and endothelial cell metabolism. The elevated circulating lipids/fatty acids/oxidized low density lipoprotein accumulate underneath the endothelial layer, creating a lipid core in the atherosclerotic plaque. Increased permeability of the endothelium via reactive oxygen species (ROS) and excess lipid accumulation leads to lipo‐toxicity, causing enhanced transmigration of immune cells through the endothelial layer. Macrophages engulf the abundant lipids, transforming into foam cells. The increased permeability and the decreased nitric oxide (crossed‐NO symbol) production from endothelial cells exacerbate endothelial dysfunction. Concomitantly, pro‐inflammatory T‐cell subsets secrete pro‐inflammatory cytokines, which further augment endothelial dysfunction in a chronic and vicious loop. The box in the upper left corner indicates the known sex differences among endothelial and immune function in physiological conditions. CMD, cardiometabolic disease; IL, Interleukin; MMPs, metalloproteinases; oxLDL, oxidized low‐density lipoprotein; ROS, reactive oxygen species; TGF‐β, Transforming growth factor beta Created with Biorender; Th1, T helper 1 cells; Th17, T helper 17 cells; Th2, T helper 2 cells; TNFα, Tumor necrosis factor alpha; Treg, T regulatory cells.

Endothelial cells (ECs) are critical regulators of vascular function, serving as a dynamic interface between the bloodstream and surrounding tissues. ECs can regulate vascular tone in response to physiological demands, by synthesizing vasodilators, such as nitric oxide (NO) and prostacyclin [21, 22], as well as vasoconstrictors, including Endothelin‐1 and Angiotensin II [23, 24]. One of the most important vasodilatory mechanisms is the NO‐driven mechanism. In ECs, NO is produced by NO synthase (eNOS) from L‐Arginine, which, together with other co‐factors such as NADPH [25], leads to the production of NO [26]. NO is essential for vasodilation, as it diffuses to the adjacent vascular smooth muscle cells (VSMCs), where it activates guanylate cyclase [27]. The consequent production of cyclic guanosine monophosphate decreases intracellular calcium on VSMCs, leading to vascular relaxation and hence vasodilation [27]. Importantly, NADPH is not only critical for NO production, but also essential to counteract oxidative stress [28]. Given the oxygen‐rich arterial environment, NADPH ‐ among other anti‐oxidant agents‐ is fundamental to limit excessive oxidative stress [29]. Oxidative stress can lead to disruption of cell–cell contact with consequent increase of EC permeability [30], expression of adhesion molecules [31], recruitment of immune cells, and initiation of pro‐inflammatory processes [32]. Importantly, NADPH production is tightly connected to the EC metabolism, which can profoundly influence endothelial function [33]. In fact, quiescent ECs mostly rely on glycolysis for ATP production [34], while proliferating ECs decrease glycolysis in favor of fatty acid oxidation [35]. NADPH production can be indirectly enhanced by glycolysis, as glucose‐6 phosphate feeds into the pentose phosphate pathway—an essential source of NADPH [36]. Altogether, the endothelium's ability to supply surrounding tissues with essential nutrients, signaling molecules, and cellular components, as well as to regulate vascular tone, is tightly dependent on its metabolic state. Vascular endothelial dysfunction is closely associated with the onset and progression of obesity and related metabolic and cardiovascular complications, such as hypertension and atherosclerosis [37]. T2D, characterized by dysregulated glucose control, strongly affects EC metabolism [38]. Indeed, excessive glucose paradoxically impairs glycolysis [39], resulting in reduced NADPH and diminished anti‐oxidant effect [39]. An imbalance between reactive oxygen species (ROS) and anti‐oxidant defenses directly damages ECs, and reduces NO availability [40]. Similarly, while at physiological levels fatty acids are normally taken up by ECs and stored in lipid droplets [41], in excessive concentrations, they cause lipo‐toxicity, increase the permeability of the endothelium, and attract immune cells, further aggravating endothelial damage and inflammation [42] (Figure 1).

### 2.2 Sex Differences in Endothelial Function

Various sex‐related differences impact endothelial function. While research exploring the role of sex chromosomes is beginning to emerge [43, 44], the most extensively studied and well‐characterized influences are those driven by sex hormones [45]. Indeed, sex hormone receptors are present in endothelium [45, 46] and in vitro experiments on different types of ECs, such as human pulmonary, aortic, coronary, and umbilical ECs, show that 17β‐estradiol enhances NO production [47, 48]. Interestingly, the in vitro effect of androgens on eNOS expression and activation in human umbilical endothelial cells (HUVECs) is observed at physiological levels of testosterone or dihydrotestosterone (DHT) (100 pmol/L‐100 nmol/L) and lost with supra‐physiological levels (> 100 nmol/L), suggesting that only a well‐defined concentration of testosterone or DHT can actually exert beneficial effects [49]. The increased NO production induced by androgens could partly reflect the conversion of androgens to estrogen by the aromatase enzyme, as this enzyme is present in ECs [49]. This conversion could, however, be specific to the microvasculature, as the aromatase enzyme is not expressed in the larger arteries, such as the aorta or the pulmonary artery [50]. 17β‐estradiol generally exerts a protective role in EC activation and the initiation of inflammation [51, 52, 53] by reducing tumor necrosis factor alpha (TNFα)‐mediated adhesion of monocytes and by reducing the production of interleukin 8 (IL‐8) and monocyte chemoattractant protein‐1 (MCP‐1) from HUVECs [51]. Interestingly, the decreased production of these pro‐inflammatory cytokines is due to their reduced secretion rather than reduced transcription, suggesting a non‐genomic action of 17β‐estradiol. These in vitro results were further confirmed in a clinical trial in which post‐menopausal women subjected to hormone replacement therapy with 17β‐estradiol display reduced MCP‐1 and adhesion molecule levels in their serum [52, 53]. Similarly, to the effects of testosterone and DHT on NO production, the effect of androgens on EC activation is also concentration‐dependent. Some studies report a reduction of TNFα‐induced vascular cell adhesion protein 1 (VCAM‐1) in HUVECs and human aortic endothelial cells (HAECs) with physiological levels of DHT and testosterone (≤ 100 nM) [54, 55]. Importantly, this reduction was mediated by the androgen receptor‐dependent transcription of nuclear factor kappa B (NFκB) [55]. On the other hand, a few studies also report increased NFκB‐dependent expression of VCAM‐1 in HUVECs and HAECs with supra‐physiological (400 nM) concentrations of testosterone [56, 57]. This divergence in androgen effect is highly concentration‐dependent, supporting the notion of a well‐controlled mechanism of action. Altogether, sex hormones modulate endothelial function in a concentration‐dependent manner: 17β‐estradiol promotes NO production and reduces inflammation, while testosterone and DHT can be protective at physiological levels but pro‐inflammatory at high concentrations.

Sex hormone‐independent influence of ECs is less studied and characterized in the field of endothelial function. A recent genome‐wide association study (GWAS) performed on ECs isolated from umbilical cords of girl‐boy twins revealed that several genes are differentially expressed in the two sexes, and the difference is independent of the sex hormones [58]. Interestingly, one of these genes encoding for an ATPase (ATP2B1) which dampens eNOS function, shows lower expression in female [58]. These results align with the experimental findings, showing greater basal protein kinase G (PKG)‐mediated NO production in female rats compared with males, resulting in a reduced constrictor response to phenylephrine [59]. Interestingly, this effect was sex hormone‐independent, as proven by ovariectomy, suggesting an involvement of sex hormone‐independent mechanisms. Additionally, higher NO production and concomitant lowering of ROS levels was also shown in a study comparing female and male HUVECs [60] (Table 1).

**TABLE 1:** Qualitative overview of the main physiological differences between healthy female (pre‐menopausal) and male in endothelial and immune cells. Created with Biorender.

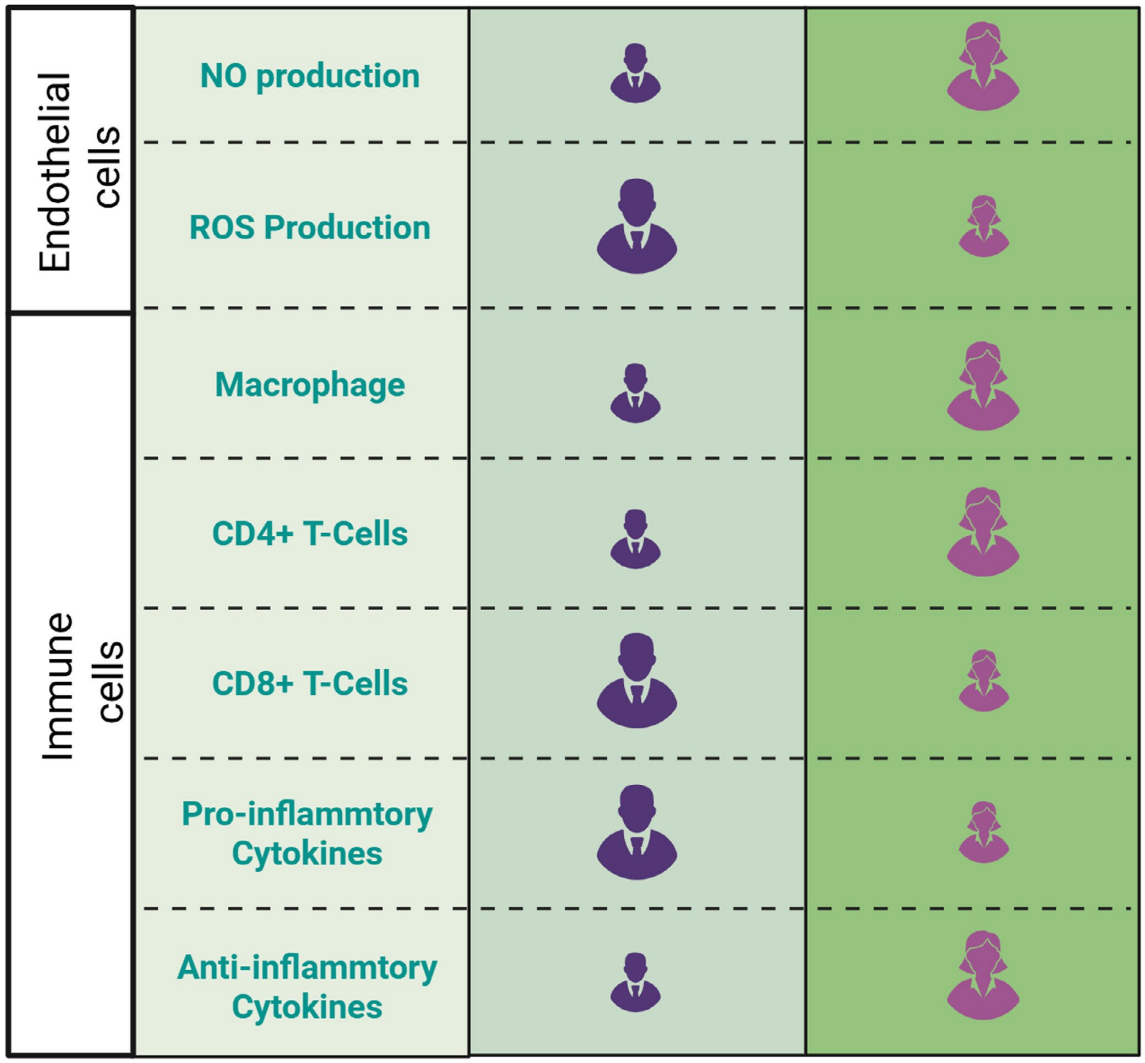

Another GWAS study identified a higher male‐biased variant of the gene encoding for endothelin receptor A and for nectin‐2, involved in endothelial vasoconstriction and trans‐endothelial migration of leukocytes, respectively [61], supporting increased vasodilation and decreased permeability in females compared to males (Table 1). DNA methylation studies revealed that men display an inactivation of cystathionine γ‐lyase due to its hypermethylation [62]. This enzyme is responsible for producing hydrogen sulphide, which acts as a vasodilator and anti‐oxidant in the endothelium [63]. Altogether, these results show a sex hormone‐independent importance of sex‐specific epigenetic mechanisms and sex‐driven genetic variance.

Altogether, these results suggest that sex hormone–independent biological differences may create an intrinsic vulnerability for CVD in men compared to women. In women, the loss of the additive protective effect of 17β‐estradiol during menopause could potentially explain the higher cardiometabolic risk in post‐menopausal women [64].

### 2.3 Immune Cell and Cytokine Function

Immune cells defend the body against pathogens, regulate tissue surveillance and repair, and maintain metabolism and inflammation [65]. A dysregulation of these physiological mechanisms contributes to the progression of all CMDs, leading to a characteristic chronic low‐grade inflammation. This occurs as a result of immune dysfunction, characterized by changes in the circulating levels of cytokines via increased inflammatory mediators like IL‐1β, IL‐6, Interferon gamma (IFN‐γ), TNF‐α, and reduced levels of cytokines like adiponectin and IL‐10, which have an anti‐inflammatory function [66]. Among several different immune cells, this section will focus on the three major homeostatic balances in immune cell phenotype that need to be maintained for healthy function.

The immune dysfunction and chronic low‐grade inflammation both result from dysregulation in the delicate balance between three pairs of pro‐inflammatory and anti‐inflammatory immune cells: M1/M2 macrophages, CD4‐positive (CD4+) type 1 helper (Th1)/Type 2 helper (Th2) and IL‐17 secreting (Th17)/regulatory T (Treg) cells [67].

#### 2.3.1 Macrophages

Macrophages are versatile immune cells that maintain tissue homeostasis, orchestrate immune responses, and facilitate repair processes [68]. Derived from monocytes or yolk‐sac progenitors during embryogenesis, macrophages are widely distributed across tissues, adapting their functions to meet the specific physiological demands of their local environments [69]. For example, macrophages contribute to embryonic development by shaping tissues and vasculature through secretion of growth factors like vascular endothelial growth factor [70]. They also have a key function in tissue injury and repair through secretion of growth factors like transforming growth factor beta (TGFβ) and platelet‐derived growth factor, which support regeneration [71].

When dysregulated, they act as key drivers of inflammation and tissue remodeling in CMD, and their dysfunction in the balance between the M1/M2 phenotypes (Table 2) perpetuates inflammation, metabolic dysregulation, and cardiovascular strain, driving the progression of diseases such as atherosclerosis and hypertension [72]. Macrophages have been extensively studied in the context of CMDs, where, in obesity and T2D a polarization occurs towards the M1 pro‐inflammatory phenotype, mainly in the adipose tissue. This shift results in a reduction of M2 phenotype macrophages, which, in turn reduce anti‐inflammatory signals while driving up pro‐inflammatory signals [73]. In atherosclerosis, circulating monocytes are recruited to atherosclerotic lesions where they activate to become macrophages and engulf oxidized low‐density lipoprotein (oxLDL), transforming into foam cells that secrete cytokines (e.g., IL‐1β, TNF‐α) and matrix metalloproteinases (MMPs), promoting inflammation, plaque instability, and potential rupture [74]. In a rat model of hypertension, macrophages caused inflammation in ECs through macrophage‐derived exosomes [75]. Macrophages contribute to intimal thickening, extracellular matrix remodeling, and fibrous cap formation through IL‐1β‐mediated interaction with VSMCs in a STAT3‐dependent manner—all processes that are hallmarks of vascular remodeling during atherosclerosis progression [76].

**TABLE 2:** Specification of different immune cell subsets.

Cell type	Abbreviation	Surface markers	Transcription factor	Secretory profile	Role in CMD
Type1 macrophages	M1	MHC‐II CD86+ CD80+	IRF3, IRF5	TNFa, IL6, IL12	Proinflammatory
T‐helper 1	Th1	CD4+ CXCR3− CCR4− CCR6+	T‐bet	IFN‐γ, TNF‐α	
T‐helper 17	Th17	CD4+ CXCR3+ CCR4− CCR6−	RORC2	IL‐17, IL‐22	
Type 2 macrophages	M2	CD163+ CD206+ CD209+	IRF4, KLF4	Arginase‐1, IL10	Anti‐inflammatory
T‐helper 2	Th2	CD4+ CXCR3− CCR4+ CCR6−	GATA‐3	IL‐4, IL‐5, IL‐13	
Regulatory T cells	Treg	CD4+ CD25+ CD127−	FOXP3	IL‐10, TGF‐β	

#### 2.3.2 CD4 ± T‐Cells

CD4 + T‐cells, also known as helper T‐cells, are a crucial subset of lymphocytes in the adaptive immune system that play a central role in coordinating and orchestrating the activity of innate and adaptive immune cells through cytokine secretion and cell–cell interactions [77]. CD4 + T‐cells secrete specific cytokines based on their differentiation into distinct subsets with specific functions (Table 2). The activation and differentiation of CD4 + T‐cells are influenced metabolically. In a naive state, T‐cells primarily rely on oxidative phosphorylation and fatty acid oxidation for ATP generation. Upon activation, they shift to glycolysis for rapid energy production, even in the presence of oxygen, following the classic Warburg effect, to support effector differentiation [78].

CD4 + T‐cells play a significant role in orchestrating inflammation, contributing to disease initiation, progression, and complications. The balance between pro‐inflammatory and regulatory subsets of CD4 + T‐cells is critical in determining the overall immune response in CMD [79]. Apart from the dysregulation of the pro/anti‐inflammatory balance in CMD, a shift towards a state of cellular dysfunction that occurs after prolonged activation is observed, termed T‐cell exhaustion [80, 81]. The persistent low‐grade inflammation and metabolic stress can drive T‐cell exhaustion, causing progressive loss of effector functions, reduced cytokine production, and impaired proliferation [82, 83]. This section discusses the CD4+ subsets in the context of obesity, T2D, and related comorbidities.

##### 2.3.2.1 Th1/Th2 Balance

Th1 cells function through the production of IFN‐γ, exacerbating tissue damage and metabolic imbalances, fueling a cycle that worsens CMD. In obesity, elevated levels of certain fatty acids (e.g., butyrate) promote Th1 differentiation in humans and mice [84, 85], while systemic metabolic stress further activates Th1 cells [86]. In mice, an imbalance in the Th1/Th2 ratio towards a pro‐Th1 phenotype leads to increased atherosclerotic progression in an IL‐6‐dependent manner, working in tandem with acute phase reactants like serum amyloid A [87]. Additionally, Th1 cells are involved in hypertension pathogenesis via IFN‐γ‐induced overexpression of angiotensin in rat renal proximal tubule cells in a JAK2/STAT3‐dependent manner [88, 89]. The IFN‐γ secreted by Th1 cells also causes activation of cardiac fibroblasts by inducing TGF‐β in mice [90], suggesting an immune‐mediated mechanism for cardiac fibrosis and dysfunction resulting in heart failure. Th2 cell responses, which normally occur through the secretion of cytokines like IL‐4, IL‐5, and IL‐13, are very context‐dependent. In obesity, Th2 cells promote downstream differentiation of M2‐macrophages via the activation of STAT6 in adipose tissue, promoting a more anti‐inflammatory profile [91]; however, chronic Th2 activation can paradoxically contribute to metabolic dysregulation by inducing fibrosis and altering tissue structure [92]. On the other hand, IL‐4 and IL‐13 can stimulate cardiac fibroblasts and inadvertently promote maladaptive tissue remodeling and fibrosis, worsening outcomes in conditions like hypertension, obesity, and heart failure [93].

##### 2.3.2.2 Th17/Treg Balance

Th17 cells contribute to CMD by producing the pro‐inflammatory cytokine IL‐17, which recruits and activates neutrophils and amplifies inflammation in affected tissues such as adipose, liver, and vascular walls [94]. In hypertension in rats, Th17 cells increase blood pressure and induce vascular dysfunction by strongly enhancing pathways that lead to an increase in salt reabsorption and elevated vascular tone [95]. At a molecular level, Th17‐mediated IL‐17 secretion via the MAPK‐NFκB signaling could promote oxidative stress and further inflammatory cytokine release contributing to hypertension [96]. Studies on rodent models of hypertension induced by Angiotensin II [95], mineralocorticoid‐dependent hypertension [97], hypertension in response to cyclosporine A [98], or in a model of pre‐eclampsia [99] with IL‐17 attenuation by genetic intervention or by receptor blockade have all shown that a lack of IL‐17 prevents hypertension. Th17 cells also exacerbate obesity‐related insulin resistance by increasing inflammation in adipose tissue [100]. In obesity, elevated IL‐6 causes the number of Th17 cells to increase. IL‐17 secreted by Th17 targets adipocytes and triggers downstream pro‐inflammatory signaling, while IL‐21 secreted by Th17 inhibits Treg differentiation and function [101]. These effects in obesity, in turn, accelerate progression to CVDs. Th17‐mediated pro‐inflammatory responses and reduction in Treg‐mediated anti‐inflammatory responses exacerbate atherosclerosis, acute coronary syndrome, and congestive heart failure [102]. In heart failure with preserved ejection fraction, elevated Th17 cell activity contributes to fibrosis and cardiac dysfunction, worsening disease progression and outcomes [103]. Treg cells are best known as critical regulators of inflammation and metabolism and function by secreting suppressive cytokines like IL‐10 and TGF‐β [104].

### 2.4 Sex Differences in Immune Function

Sex differences in immune responses are influenced by genetic, hormonal, and environmental factors. The X chromosome carries numerous immune‐related genes, including those encoding Toll‐like receptors (e.g., TLR7), cytokine receptors, and immune signaling molecules [105]. The Y chromosome encodes male‐specific factors that modulate immunity, such as male‐specific minor histocompatibility antigens [106]. Sex hormones modulate immune responses by influencing the activity of immune cells, driving stronger immunity against infections in women (via estrogens) and suppressing inflammation in men (via androgens) [107]. These factors together contribute to females typically having more robust macrophage activation (Table 1) and cytokine production, such as TNF‐α, IL‐1β, and IL‐6, compared to males. Women also have a higher CD4 + T‐cell count and greater cytokine production (e.g., IL‐4, IL‐10), favoring Th2 responses [108]. Men exhibit a higher proportion of CD8^+^ T‐cells but with reduced cytotoxicity and persistence [108] (Table 1).

Sex differences therefore play a crucial role by driving differences in immune cell populations. In fact, macrophages and T‐cells and have diverse functions, contributing to sex‐specific CMD development by mediating inflammation, tissue remodeling, and metabolic regulation.

17β‐estradiol in pre‐menopausal women generally enhances anti‐inflammatory immune functions. It promotes regulatory Treg activity, which helps suppress excessive inflammation by releasing cytokines like IL‐10 and TGF‐β, supporting tissue tolerance and immune homeostasis [109]. Differences in levels of 17β‐estradiol can also affect the balance between Th1/Th2 cell types, where lower levels (luteal phase) encourage a skew towards the Th1 phenotype via IFN‐γ [110] and higher levels (follicular phase) promote Th2 differentiation through increased IL‐4 production [111]. 17β‐estradiol also pushes macrophages to adopt an M2 phenotype, which is associated with tissue repair and anti‐inflammatory effects [112]. Pre‐menopausal women have a lower prevalence of atherosclerosis and hypertension than age‐matched men [113] which could be partly attributed to this estrogen‐mediated immune modulation, providing a degree of protection against CMD‐related inflammation in women [114]. However, after menopause, with declining 17β‐estradiol levels, Tregs and M2 macrophages decrease, and inflammatory T‐cell populations like Th1 and Th17 increase, resembling the more pro‐inflammatory immune environment typically seen in men. This shift corresponds to a heightened CMD risk in post‐menopausal women [115].

In clinical studies, testosterone provides anti‐inflammatory effects via the suppression of pro‐inflammatory cytokines and the simultaneous enhancement of anti‐inflammatory cytokines [116]. In vitro studies using peripheral blood mononuclear cells from humans treated with anabolic androgen (metribolone) and in vivo studies using orchiectomized animal models have shown that androgens inhibit Th1 and potentially Th17 differentiation through up‐regulation of phosphatase Ptpn1 [117], while causing (DHT‐treated CD4 + T‐cells) an expansion of Treg by upregulating FOXP3 expression [118]. Several correlation studies have shown that endogenous testosterone levels in men declines with age, while cardiovascular mortality increases [119, 120, 121, 122]. Though at physiologically optimal levels, testosterone is considered cardioprotective, one study has shown that, similarly to high testosterone levels, low levels of testosterone are also correlated with an increased risk of ischemic arterial events [123]. The effects of testosterone on CMD could be through modulation of immune cells, but this needs further understanding. Overall, these sex‐based immune differences affect CMD progression, with women showing a greater predisposition towards autoimmune diseases [124], and men showing testosterone dose‐dependent CMD phenotypes. Understanding these mechanisms may improve precision treatments, considering sex‐specific immune responses to address CMDs more effectively.

## 3 The Incretins in Physiology and Disease

### 3.1 Incretin and Incretin‐Analog Biology

Incretins are members of the glucagon superfamily and are gut‐derived hormones that play an important role in whole‐body physiology. There are two main incretin hormones in humans, namely GLP‐1 and GIP. GLP‐1 is derived from the proglucagon‐preprotein (encoded by the GCG gene) and processed into its active form through multiple cleavage steps [125]. This occurs mostly in specialized epithelial cells called enteroendocrine L‐cells, located within the mucosal lining of the ileum and colon in the gastrointestinal tract [126], and only in a minor percentage in the pancreatic α‐cells [127, 128, 129, 130]. GLP‐1 is secreted in response to carbohydrate and fat intake to enhance insulin secretion from pancreatic β‐cells in a glucose‐dependent manner. Moreover, GLP‐1 inhibits glucagon release and slows gastric emptying, promoting satiety and reducing food intake [131, 132, 133]. Additionally, GLP‐1 enhances browning of white adipose tissue, increases intracellular cholesterol efflux in macrophages, and also limits steatosis in the liver [134, 135, 136]. GIP is produced in K‐cells of the small intestine; it is similarly stimulated by nutrient ingestion, particularly carbohydrates and fats, and functions by controlling nutrient balance and blood glucose levels [10]. GIP may stimulate glucagon release in certain conditions such as T2D [137] and plays a role in lipid metabolism by promoting triglyceride clearance from blood and increasing fat storage in adipocytes [138, 139]. GLP‐1 and GIP carry out their signaling through G‐protein coupled receptors: GLP‐1R and GIPR, respectively. In the blood circulation, GLP‐1 and GIP both have very short half‐lives of just a few minutes, being degraded by dipeptidyl peptidase‐4 (DPP4)‐mediated proteolysis which is the mechanism that maintains incretin hormone homeostasis [140].

The therapeutic use of incretin mimetics, particularly GLP‐1R agonists and dual agonists to GLP‐1R/GIPR, has gained prominence in the last decade. The agonists enhance insulin secretion in a glucose‐dependent manner, suppressing glucagon release, and promoting weight loss by increasing satiety [9, 141, 142]. Incretin‐based therapy exerts effects such as reducing body weight (BW) by influencing appetite in the brain [143] as shown in a clinical trial with semaglutide vs. placebo [144] Additionally, incretin‐based therapy decreases adipose tissue mass and enhances EC vasodilation, as evidenced by a clinical trial with exenatide in male patients with hypertension and other cardiovascular complications associated with obesity and T2D [145]. The effect of co‐administration of GIPR and GLP‐1R agonists in rodents has shown enhanced effects on lowering blood glucose and promoting weight loss [146]. Tirzepatide, an imbalanced dual agonist of GLP‐1R/GIPR, has been approved for therapy in both T2D [147] and obesity [148]. Clinical trials with tirzepatide have shown a greater effect on glycemic control through improving β‐cell function while reducing glucose excursions, in terms of lowering insulin and glucagon secretion, compared to single GLP‐1R agonist therapy, supporting the beneficial role of the GIPR agonist [149]. On a molecular level in human pancreatic cells, tirzepatide has high affinity for GIPR‐based signaling while a lower affinity towards GLP‐1R [150]. This mechanism leads to internalization of the GIPR, working effectively as a functional antagonist of GIP instead of its intended agonistic purpose [151]. Along this notion, an ongoing phase 2 clinical trial employing GLP‐1R agonist/GIPR antagonists (MariTide, NCT05669599) has also shown effective reduction in BW and better glycemic control compared to the placebo arm. Altogether, the exact mechanisms by which GIPR‐targeting drugs exert their effects remain unclear—particularly whether they act through receptor agonism or antagonism [152]—highlighting the need for further research.

### 3.2 Effects of Incretins in Endothelial Cells

GLP‐1R and GIPR are present on venous and arterial vascular ECs [153, 154], enabling direct actions of hormones or agonists. Indeed, GLP‐1, but not GIP, increases NO production in HUVECs through cAMP signaling [153]. Similarly, the GLP‐1R agonist exendin activates the PI3K/Akt –eNOS pathway in human coronary artery ECs (HCAECs), resulting in increased proliferation as well [155]. An in vitro study using palmitate‐induced oxidative injury in islet microvascular ECs shows that liraglutide treatment ameliorates oxidative injury in a PKA/eNOS‐dependent manner [156]. In line with this study, in vivo, GLP‐1R activation by liraglutide preserved endothelium‐mediated vasorelaxation in Angiotensin II‐induced hypertensive rats [154]. In this study, the preserved NO bioavailability in liraglutide‐treated rats resulted from decreased oxidative stress and prevented uncoupling of eNOS due to a lower vessel wall infiltration of inflammatory monocytes and neutrophils [154]. Corroborating the anti‐inflammatory effect of GLP‐1 and its analogues, another study showed that GLP‐1 limited the advanced glycation end product (AGE)‐induced up‐regulation of VCAM‐1 expression in hyperglycemic HUVECs by suppressing the receptor of AGE (RAGE) and the subsequent generation of ROS [157]. However, not only the anti‐inflammatory effects induced by GLP‐1 are PI3K/Akt/eNOS‐mediated, but also its angiogenic potential. Indeed, recent studies demonstrate that GLP‐1 promotes angiogenesis not only in vitro in HUVECs [158] but also in vivo in a model of diabetic hind‐limb ischemia [159]. In both studies, the angiogenic effect was mediated by the Akt/eNOS pathway, suggesting a crucial role of this pathway in many GLP‐1‐induced effects. GLP‐1‐mediated pathways have also been implicated in reversing endothelial dysfunction in obese rats [160] and in enhancing coronary artery vasodilation in humans [161]. On the other hand, in some studies, GIP increases Endothelin‐1 [162, 163, 164] and also NO [160], production, while other evidence documents no influence of GIP on NO production [151]. These differences could be partially attributed to variations in EC sources, such as arterial versus venous origins [162], or distinct compartments, like liver arteries versus umbilical veins [153]. However, the exact mechanism driving these discrepancies remains unclear. Altogether, these results clearly suggest a vaso‐protective effect of GLP‐1 analogs while GIP‐targeted therapies are less studied and shown to be more varied and context‐dependent.

### 3.3 Effect of Incretin in Immune Cells

Since the advent of incretin‐based therapy and its anti‐inflammatory impact, there is increasing attention given to the interaction between incretin hormones and immune cells that is not fully understood. Both GLP‐1R and GIPR are expressed on several immune cell subsets, like CD4+/CD8+ T‐cells [165, 166] and macrophages [166, 167]. GLP‐1 increases cholesterol efflux in macrophages by increasing ATP‐binding cassette transporter A1 (ABCA1) or apolipoprotein AI (apo AI) expression [135]. A study conducted using a GLP‐1R knockout mouse model has shown that GLP‐1R signaling may regulate lymphocyte proliferation and maintenance of peripheral regulatory T‐cells [168]. Under metabolic stress, GIP and its receptor link energy availability to the control of hematopoiesis by modulating bone marrow TLR and Notch signaling [166]. GLP‐1R is shown to act as a negative co‐stimulatory molecule in a subset of CD4+ and CD8+ T‐cells, controlling T‐cell activation, IFN‐γ secretion, cell metabolism, and death [165]. Both GLP‐1 and GIP have been implicated in the regulation of Th17/Treg balance, which is essential for immune homeostasis, in many diseases such as inflammatory bowel disease, gastrointestinal cancers, and several autoimmune diseases [169, 170]. The exact mechanisms of GLP‐1 and GIP receptor signaling remain unclear; however, the adoptive transfer of GIPR‐deficient bone marrow into high‐fat diet‐fed mice reduces Treg populations in visceral adipose tissue compared to wild‐type bone marrow [171]. Additionally, the GLP‐1 analog liraglutide selectively inhibits Th1 and Th17 proliferation in vitro, indicating an anti‐inflammatory effect [172]. Studies using the agonists have illuminated the direct immuno‐metabolic and immuno‐regulatory roles of GLP‐1 and GIP on multiple immune cell subsets [173]. Indeed, incretin agonists have a significant effect on the underlying chronic inflammation in CMDs [174] and on the general metabolism of immune cells [18]. GLP‐1 and GIP reprogram immune cell metabolism by shifting macrophages [175] and T cells from a pro‐inflammatory, glycolysis‐driven state towards oxidative phosphorylation and lipid metabolism. This metabolic rewiring supports anti‐inflammatory phenotypes, including M2‐like macrophages [175] and regulatory or anergic/exhausted T cells, contributing to immune tolerance and reduced tissue inflammation [165]. The effects of the agonists are mostly anti‐inflammatory and of a protective nature. Specifically, 12 weeks of treatment with GLP‐1R agonists in T2D patients (data about the sex of participants are not reported) reduced inflammation significantly by reducing the production of ROS, inflammatory mediators like TNF‐α/IL‐1β, and NFκB activation in blood mononuclear cells [176]. GLP‐1R agonism is also known to directly inhibit monocyte adhesion to ECs, leading to an attenuation of the atherosclerotic plaque [177]. In murine models of obesity, liraglutide could effectively change the CD4 + T‐cell subsets and the cytokines, leading to an increase in Th2 and Treg and a concomitant reduction in Th1 and Th17 cells [178]. The role of GIP, on the other hand, is context‐dependent. Paradoxically, both agonism and antagonism of GIPR can either exacerbate or alleviate inflammation [179]. The dual agonist tirzepatide alleviates oxidative stress and IL‐17‐based inflammation [180].

### 3.4 Sex Differences on Incretin Biology and Therapy

A significant amount of research has focused on identifying the exact mechanisms of incretin action. However, fewer mechanistic biological or animal studies have explored potential sex‐related differences in the efficacy of incretin‐based treatments. In clinical trials, although both sexes are typically included, females are often underrepresented, making it challenging to conduct a proper comparison between the sexes [181, 182] and to draw reliable conclusions for both sexes. However, a recent study conducted in T2D patients demonstrated that hazard ratios (HRs) for cardiovascular events (non‐fatal acute myocardial infarction, unstable angina, heart failure, or stroke) were lower with GLP‐1RA compared with sulfonylureas, in women than in men [183], which could be attributed to the greater weight loss achieved in females compared to males [184]. The physiology of incretin shows differences between men and women regarding production and effects. Indeed, pre‐menopausal women exhibited a 2‐fold enhanced GLP‐1 and insulin production as measured in the circulation during standardized intraduodenal glucose (2 and 3 Kcal/min) injection compared to men [185]. The increased GLP‐1 production in females is further enhanced in the luteal phase of the menstrual cycle compared to the follicular phase [186], suggesting also an association of GLP‐1 production with progesterone. Estrogen also influences GLP‐1 secretion. Ovariectomized reporter mice (Venus fluorochrome in proglucagon‐producing cells and the Cherry fluorochrome in insulin‐producing cells) present decreased GLP‐1 production, which could be reversed with 17β‐estradiol supplementation [187]. Similarly, 17β‐estradiol enhances GLP‐1 release from human pancreatic α cells [187]. Importantly, this effect is mediated by both genomic (estrogen receptor α/β (ERα and ERβ)) and non‐genomic pathways (G protein‐coupled estrogen receptor 1, GPER‐1) in the pancreas but only by the genomic ERβ signaling in the intestine [187]. Estradiol not only directly influences GLP‐1 production and action, but can have an additive effect on GLP‐1 action. Indeed, a stable GLP‐1‐17β‐estradiol conjugate has demonstrated superior efficacy over GLP‐1 single hormone in treating metabolic syndrome in both male and female mice [188]. Interestingly, the GLP‐1‐17β‐estradiol conjugate was able to synergistically activate GLP‐1‐responsive anorexic regions in the central nervous system, leading to a higher reduction in hunger compared to GLP‐1 alone [189, 190]. This effect was mediated by GLP‐1 functioning as a carrier to deliver 17β‐estradiol into GLP‐1 receptor–expressing cells, where it could activate estrogen‐responsive elements [191]. The clear synergistic effect of estrogen and GLP‐1 could explain some sex‐associated effects observed in incretin therapy. For instance, dulaglutide induces greater BW loss in women (post‐menopausal) compared to men [184, 192]. Additionally, more severe gastrointestinal adverse effects were also observed in women compared to men [184], a result which was attributed to a higher plasma concentration of the drug due to lower BW of women compared to men. However, a subsequent study revealed that at equal plasma concentrations of liraglutide, women (pre‐ and post‐menopausal) still experienced more gastrointestinal adverse effects [193]. These results suggest a clear synergistic positive effect of estrogen and incretins, which are further complicated in transgender medicine. Indeed, a recent study has revealed decreasing insulin sensitivity in transgender women (male to female) following hormonal therapy [194], clearly indicating a negative effect of female hormone therapy on biological men. On the other hand, the same study reports increasing insulin sensitivity in transgender men (female to male) following male hormonal therapy, suggesting a positive effect of male hormonal therapy on biological females. This observation is in line with a mechanistic study that reported a positive effect of DHT in enhancing GLP‐1‐mediated insulin production from pancreatic β‐cells [195]. The relationship between androgens and incretins might, however, be bidirectional. Indeed, a pilot prospective randomized open‐label study observed a normalization of testosterone levels upon liraglutide treatment in hypogonadal obese men [196]. On the other hand, liraglutide in combination with metformin was shown to decrease testosterone levels in obese polycystic ovary syndrome patients in comparison to metformin alone [197].

Altogether, the current evidence clearly suggests a bidirectional influence between sex and incretin therapy; however more mechanistic studies on sex‐related differences in incretin therapy will undoubtedly be needed for a deeper understanding.

## 4 Interplay and Outlook

The reviewed literature shows the close connection existing in CMDs between vascular and immune dysfunction and metabolic impairments. Dysmetabolic ECs undergo heightened glycolysis at first and then stalled glycolysis later, leading to oxidative stress, increased EC permeability/activation, and decreased NO production [198]. CMDs are associated with low‐grade inflammation driven by activated and pro‐inflammatory T‐cells, which, in a vicious loop, further exacerbates endothelial dysfunction [199]. Activated T‐cells increase glycolysis and mitochondrial oxidation, which, in the long term leads, to mitochondrial dysfunction and cell exhaustion [200]. Th17 cells exhibit their pro‐inflammatory phenotype by exhibiting active glycolysis and the pentose phosphate pathway [201], while the Tregs depend more on fatty acid oxidation and OXPHOS [202]. Interestingly, loss of Y chromosomes is tightly associated with exhaustion and senescence of immune cells, and it has been reported to increase cardiovascular risk [203], together suggesting that the sex‐variable as a key modulator of CMD. Not only the Y chromosomes but also testosterone levels, when dysregulated, seem to influence both endothelial and immune cell dysfunction [204]. This evidence suggests that alterations in sex hormones and chromosomes likely predispose individuals to endothelial and immune dysfunction. In CMD, both previously mentioned cell types are influenced by intrinsic metabolic dysfunction. Therefore, it is crucial to examine the complex interplay between ECs and immune cells in a sex‐dependent manner to deepen our understanding and help tailor more effective incretin‐based therapies.

**FIGURE 2:**
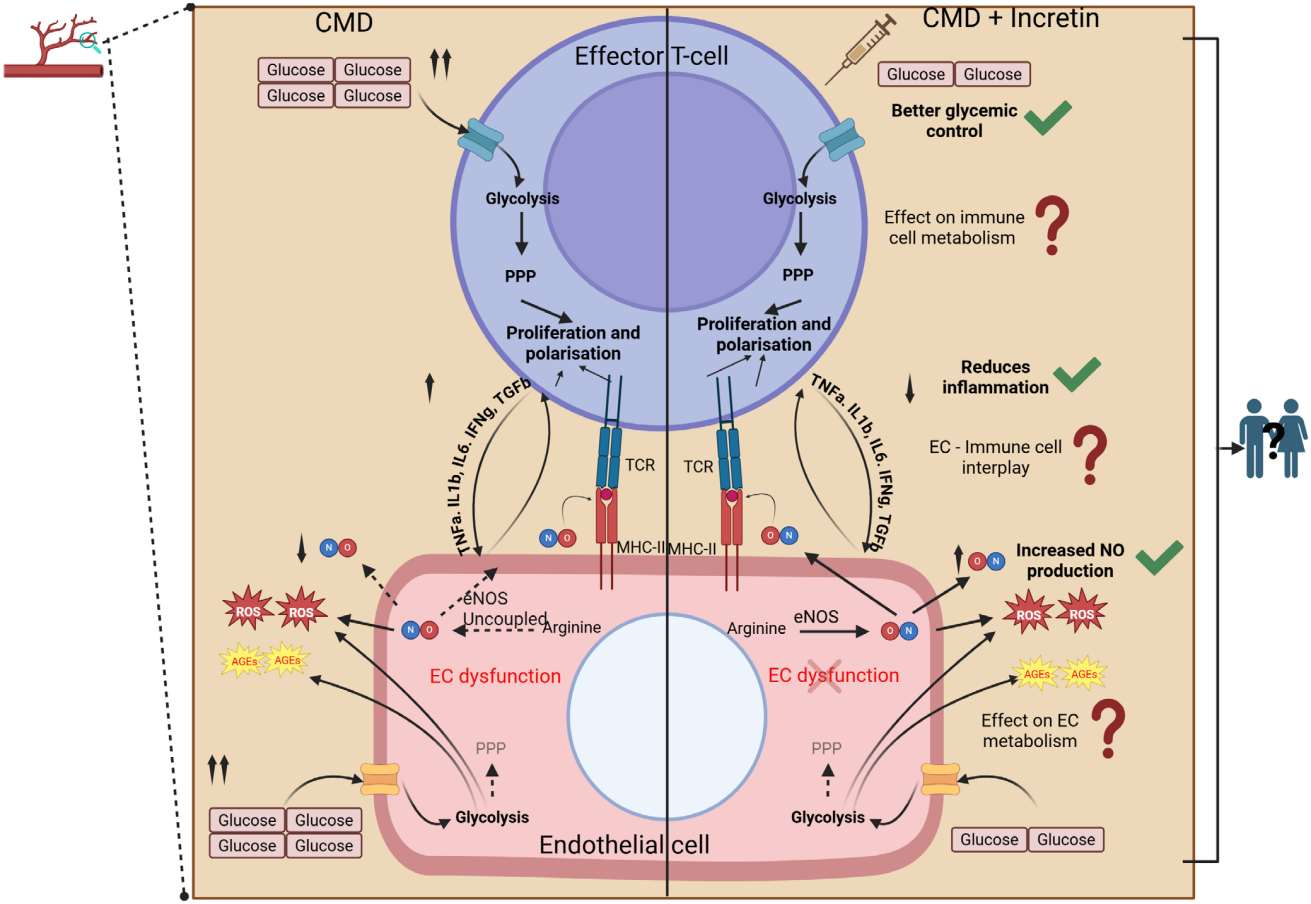
Graphical representation of specific molecular mechanisms activated in CMD at both immune and endothelial cell level. The left panel show the pathological situation, without therapy, which is characterized by increased circulatory glucose levels, EC dysfunction, eNOS uncoupling resulting in abrogation of NO production and increased inflammatory markers affecting the interaction between ECs and effector T‐cell. The right panel represents the current knowledge about incretin therapy in terms of its effect on reduction of circulatory glucose levels, reduction in inflammatory cytokine production and restoration of EC function and NO production. The future questions which need to be addressed in terms of incretin therapy include the effect of incretin therapy on EC and T‐cell metabolism and the interaction between the two cells. A major open aspect of these questions is the effect of sex differences on both the already documented effects and on possible effects that could be uncovered in the future. AGEs, advanced glycation end products; CCL2, chemokine (C‐C motif) ligand 2; CMD, cardiometabolic disease; EC, endothelial cell; NO, Nitric oxide; PPP, pentose phosphate pathway; ROS, reactive oxygen species. The normal arrows show mechanisms that are intact while the dotted arrows indicate compromised/abrogated mechanisms, The upward pointing arrow show an increase in production and the downward pointing arrows show a decrease in production. The green tick marks show effects supported by evidence in literature, while the red question marks indicate the open questions. The X‐mark on EC dysfunction indicates a return from dysfunctional state to a quiescent state.

While some studies report an anti‐inflammatory effect (Figure 2), the exact mechanisms and whether incretins can rewire the dysfunctional metabolism of immune cells remain unclear. Similarly, although incretins improve endothelial function (Figure 2), it is not yet known if this is due to a metabolic shift in EC towards a quiescent phenotype. Given the significant metabolic effects of incretin analogs, it is likely that their benefits are partly mediated by direct actions on both endothelial and immune cells, as well as their interaction. It should not be forgotten that these effects are also likely indirect, as a result of weight loss and general improvement in metabolic health. In current clinical trials of incretin‐based therapies, high‐sensitivity C‐reactive protein and flow‐mediated vasodilation are the most commonly used readouts to assess immune and endothelial function, respectively (Table 3). At the cellular level, most studies focus on macrophages in the endothelial‐immune crosstalk in CMD, with little research focusing on other cell types, such as T‐cells, in the context of incretin therapies. As ECs and immune cells can cyclically activate each other [205], incretin effects on one component may indirectly influence the other.

**TABLE 3:** Summary of recent clinical trials investigating different incretin‐based therapies with specific vascular and/or immune objectives.

PMID	Authors	Year	Intervention	Population	Primary outcome	Vascular readout	Immune readout
34542221	Wilson J.M. et al.	2021	Tirzepatide vs. Placebo	T2D for 6 months or longer	Improved glycemic control and loss of BW	↓ ICAM	↓ hsCRP
30759365	Patti A.M. et al.	2019	Exenatide vs. Placebo	T2D	Improved cardio‐metabolic parameters	↑ FMD	N.A.
31326727	Anholm C. et al.	2019	Liraglutide vs. placebo	CAD + T2D (metformin +)	Decreased low density lipoprotein	N.A.	↓ hsCRP
37883939	Stenlid R. et al.	2023	Exenatide vs. Placebo	Obesity Adolescents	Reduced obesity related inflammation	N.A.	↓ IL‐18Ra
33830637	Bray J.J.H. et al.	2021	Meta‐analysis of multiple GLP‐1RAs	Pre‐diabetes and T2D	Anti‐inflammatory and anti‐oxidant effects	↓ MDA	↓ TNFα and hsCRP
35370959	Yuhan Wang et al.	2022	Meta‐analysis of anti‐diabetic drugs	T2D w/wo high CVD risk	GLP‐1Ras‐ improved endothelial function	↑ FMD	N.A.
30622967	Batzias K. et al.	2018	Meta‐analysis of anti‐diabetic drugs	T2D	GLP‐1Ras‐ improved vascular function	↓ PWV	N.A.

Abbreviations: BW, body weight; CAD, coronary artery disease; CVD, cardiovascular disease; FMD, flow mediated dilation; hs CRP, high‐sensitive c‐reactive protein; MDA, malondialdehyde; N.A., not assessed; PWV, pulse wave velocity; T2D, type 2 diabetes.

Endothelial and immune cells are influenced by sex‐dependent factors, highlighting the need to study incretin efficacy in relation to hormonal status. Hypogonadic men or post‐menopausal women may experience lower efficacy of incretin therapy due to more pronounced endothelial/immune dysfunction associated with low circulating hormone concentrations. Incretin therapies may result in greater BW loss in women, even with the same body mass index as men [184]. This difference has been attributed to interactions between incretins and sex‐specific hormones such as leptin and sex steroids [192] Interestingly, incretin hormones also exert anorexigenic effects on the brain, which has been shown to be enhanced by estrogen in synergy with GLP‐1 [189, 190], suggesting that pre‐menopausal women may benefit more from incretin therapy. While the differences in BW loss between sexes may reflect population characteristics, the therapy efficacy could vary by sex and reproductive hormonal stage. Future studies should incorporate these variables to better understand mechanisms, tailor therapies, and predict responses, especially for patients with hormonal imbalances like polycystic ovary syndrome.

Experimental and clinical studies on sex‐driven differences face key limitations, particularly the underrepresentation of females. Clinical trials should have sufficient female participants for meaningful analysis, while preclinical, in vivo studies must incorporate female mice to identify sex‐based treatment differences. Traditional animal models employed various intervention strategies such as ovariectomy, orchiectomy, hormone replacement, or receptor knockout to explore sex hormones‐induced effects but largely ignored the sex chromosomal components. To allow the differentiation between chromosomal and sex hormonal effects, the Four Core Genotype model was additionally developed [206]. Also, in vitro experiments should use both female and male cells to examine molecular sex‐dependent differences. Addressing these factors, often overlooked, should be required by funding agencies, publishers, and policymakers to advance personalized medicine.

In conclusion, while incretin therapies hold promise for treating CMD by improving cardiovascular health and immune responses, further research is needed to understand their endothelial/immune effects and the role of sex differences. Future studies should focus on the molecular and hormonal interactions underlying these effects, as well as long‐term outcomes and personalized treatments based on sex, genetics, and disease state. Addressing these gaps will help optimize incretin‐based therapy for both male and female patients, improving cardiovascular outcomes across diverse populations.

## Author Contributions

A.S.M. and V.B. contributed equally to the conceptualization, literature search, data analysis, and drafting of the manuscript and are both recognized as co‐first authors. D.Z. assisted with the critical revision of the manuscript for important intellectual content and contributed to refining the final version. E.O. provided overall guidance throughout the writing process and finalized the manuscript for submission. All authors have read and approved the final version of the manuscript.

## Conflicts of Interest

The authors declare no conflicts of interest.

## Data Availability

Data sharing not applicable to this article as no datasets were generated or analysed during the current study.
